# Sodium Tanshinone IIA Sulfonate Attenuates Scopolamine-Induced Cognitive Dysfunctions via Improving Cholinergic System

**DOI:** 10.1155/2016/9852536

**Published:** 2016-07-31

**Authors:** Qing-Qing Xu, Yi-Jun Xu, Cong Yang, Ying Tang, Lin Li, Hao-Bin Cai, Bo-Nan Hou, Hui-Fang Chen, Qi Wang, Xu-Guang Shi, Shi-Jie Zhang

**Affiliations:** ^1^Institute of Clinical Pharmacology, Guangzhou University of Chinese Medicine, Guangzhou 510405, China; ^2^The First Affiliated Hospital of Guangzhou University of Chinese Medicine, Guangzhou University of Chinese Medicine, Guangzhou 510405, China; ^3^School of Chinese Materia Medica, Guangzhou University of Chinese Medicine, Guangzhou 510405, China

## Abstract

Sodium Tanshinone IIA sulfonate (STS) is a derivative of Tanshinone IIA (Tan IIA). Tan IIA has been reported to possess neuroprotective effects against Alzheimer's disease (AD). However, whether STS possesses effect on AD remains unclear. This study aims to estimate whether STS could protect against scopolamine- (SCOP-) induced learning and memory deficit in Kunming mice. Morris water maze results showed that oral administration of STS (10 mg/kg and 20 mg/kg) and Donepezil shortened escape latency, increased crossing times of the original position of the platform, and increased the time spent in the target quadrant. STS decreased the activity of acetylcholinesterase (AChE) and increased the activity of choline acetyltransferase (ChAT) in the hippocampus and cortex of SCOP-treated mice. Oxidative stress results showed that STS increased the activity of superoxide dismutase (SOD) and decreased the levels of malondialdehyde (MDA) and reactive oxygen species (ROS) in hippocampus and cortex. In addition, western blot was carried out to detect the expression of apoptosis related proteins (Bcl-2, Bax, and Caspase-3). STS upregulated the protein expression of Bcl-2 and downregulated the proteins expression of Bax and Caspase-3. These results indicated that STS might become a promising therapeutic candidate for attenuating AD-like pathological dysfunction.

## 1. Introduction

Alzheimer's disease (AD) is one of the most prevalent neurodegenerative diseases characterized as progressive impairment of cognition and affective disorder. Amyloid plaques, neurofibrillary tangles, and neuronal death are regarded as the basic pathological abnormalities in AD [1]. Although the exact mechanisms of AD remain unclear, abundant studies indicated that oxidative stress and cholinergic dysfunction were important in the procession of causing AD [2]. Oxidative stress has been reported to lead to cell death via apoptosis [3] and degeneration of cholinergic nervous system, which result in impairments of cognition and memory [4]. Besides, Nathan et al. found that reduction of acetylcholine (ACh) level caused by disorders of some neurotransmitters occurred in AD patients [5]. Previous studies have clearly indicated that enhanced level of ACh leads to functional improvement of central cholinergic synapses and protection of neuronal degeneration [6]. Elevation of ACh achieved by inhibiting acetylcholinesterase (AChE), metabolizing enzyme of ACh, could improve the cholinergic dysfunction of AD [7]. Several AChE inhibitors, tacrine, donepezil, galantamine, rivastigmine, and memantine, have proven to improve cognitive deficits. However, these drugs are not ideal for clinical use due to their side effects, such as hepatotoxicity and adverse gastrointestinal effects [8, 9]. Therefore, it is critical to discover alternative drugs with cholinomimetic and antioxidative activities for the treatment of AD [10].

**Figure 1:**
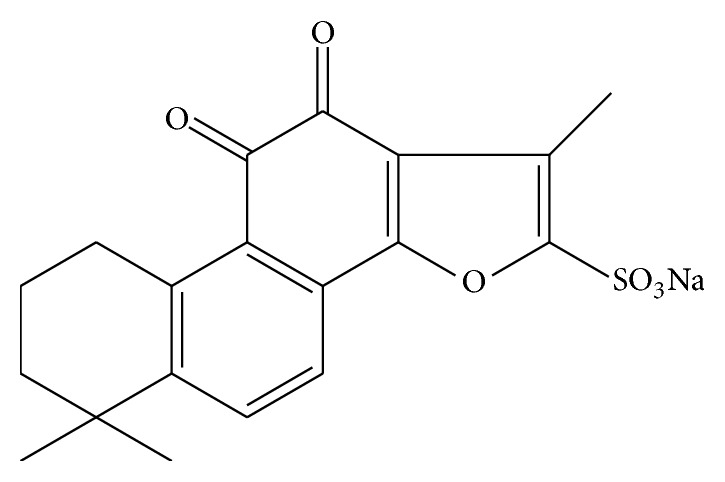
Molecular structure of STS.

Sodium Tanshinone IIA sulfonate (STS, structure shown in Figure 1) is a derivative of Tanshinone IIA (Tan IIA), which was extracted from* Salvia miltiorrhiza* [11]. Both STS and Tan IIA have well-established cardioprotective effect on cardiovascular injury [12]. In addition, Tan IIA also possesses neuroprotective activity against neural dysfunction [13]. A recent report suggested that Tan IIA can inhibit amyloid formation, disassemble A*β* fibrils, and protect SH-SY5Y and PC12 cells from A*β*-induced toxicity [14, 15]. Moreover, it has been proven that the protective effects of Tan IIA against memory deficits induced by streptozotocin (STZ) are by attenuating oxidative damage and improving central cholinergic neurotransmission [16]. Some studies show that STS and Tan IIA might have similar pharmacological actions, such as antioxidative stress [13, 17], anti-inflammation [17, 18], and antiapoptosis [19, 20]. Thus, we speculate that STS also has the protective effect on neurons.

In the present study, we evaluate the effect of STS (10 mg/kg and 20 mg/kg) on scopolamine- (SCOP-) induced learning and memory impairment in Kunming mice. Donepezil, a reversible AChE inhibitor, was employed as a positive reference drug for the treatment of cognitive deficits. Our study found that STS could obviously ameliorate cognitive impairment through attenuating oxidative damage and improving central cholinergic neurotransmission. Meanwhile, STS could protect against the apoptosis induced by SCOP in the hippocampus and cortex. These results demonstrated that STS could be served as a promising therapeutic candidate drug for AD.

## 2. Materials and Methods

### 2.1. Materials

Sodium Tanshinone IIA sulfonate (99.5%) was obtained from Loki's Pharmaceuticals (Beijing, China). Scopolamine hydrobromide injection (Guangzhou Baiyun Mountain Mingxing Pharmaceutical Co., Ltd., Guangzhou, China) was purchased from Guangzhou Pharmaceuticals Corporation (Guangzhou, China). Kits used for detection of reactive oxygen species (ROS), malondialdehyde (MDA), superoxide dismutase (SOD), choline acetyltransferase (ChAT), and acetylcholinesterase (AChE) were purchased from the Nanjing Jiancheng Bioengineering Institute (Nanjing, China). Primary antibodies (Bcl-2, Caspase-3) were purchased from Cell Signaling Technology, Inc. Anti-Bax antibody was purchased from Santa Cruz Biotechnology, Inc. Anti-*β*-actin was purchased from Sigma-Aldrich. Secondary antibodies (horseradish peroxidase conjugated anti-rabbit IgG and anti-mouse IgG) were purchased from Cell Signaling Technology, Inc. All other reagents were of the highest grade available commercially.

### 2.2. Animal and Treatment

Male Kunming mice (KM, weighing 35–40 g) were purchased from the Experimental Animal Center of Guangzhou University of Chinese Medicine (Guangzhou, China). They were maintained on standard laboratory conditions with free access to water and food. Procedures for animal experiments were conducted in accordance with the Guiding Principles for the Care and Use of Laboratory Animals as adopted and promulgated by the United States National Institutes of Health. Mice were randomly divided into five groups: vehicle control group (CON, 0.9% saline, *n* = 10), scopolamine group (SCOP, *n* = 10), low dose STS group (STSL, SCOP 3 mg/kg + STS 10 mg/kg, *n* = 10), high dose STS group (STSH, SCOP 3 mg/kg + STS 20 mg/kg, *n* = 10), and Donepezil group (DON, SCOP 3 mg/kg + ARI 3 mg/kg, *n* = 10). Mice were treated with saline, STS, and Donepezil, respectively, by gavage, once per day for two weeks. SCOP was injected from the eighth day for one week (intraperitoneally, IP). The SCOP was injected 0.5 h before the Morris water maze test.

### 2.3. Morris Water Maze Test

The Morris water maze test was similar to the method of Morris [21], with minor modifications [22]. The water maze equipment (Guangzhou Feidi Biology Technology Co., Ltd., Guangzhou, China) consisted of a black circular pool, a black platform, and a record system. The water (30 cm in depth; temperature: 22–26°C) in pool (diameter: 120 cm; height: 40 cm) was dyed with nontoxic soluble black colored. And the pool was divided into four equal quadrants. The black escape platform (diameter: 10 cm, 1.5 cm below the water surface) was placed in the center of one of the pool quadrants. The learning and memory ability of mice was detected by the Morris water maze test in a dark room. Mice were given a place navigation test for four consecutive days. For each daily trial, there were four sequential training trials beginning with placing the animal in the water facing the wall of the pool with drop location changing for each trial; then the record system starts to record the time. The escape latency was recorded at the end. If the mouse failed to find the platform within 60 s, it would be guided to the platform by the trainer and to remain there for 10 s; its escape latency would be recorded as 60 s. On the fifth day, the mice were allowed to swim freely in the pool for 60 s without the platform. The times of crossing through the original platform position and the time spent in the target quarter were measured, which indicated the degree of memory consolidation.

### 2.4. Measurement of AChE and ChAT Activity

All mice were anesthetized and decapitated after the Morris water maze test immediately; hippocampus and cortex were carefully dissected from brains for examination. All the processes were performed on ice-cold plate. Tissues were rapidly stored at −80°C. The hippocampus and cortex tissues were homogenized with ice-cold saline. The homogenate was centrifuged at 12,000 ×g for 10 min at 4°C. The supernatant was used to detect the activity of ChAT and AChE according to the manufacturer's instructions by using Universal Microplate Spectrophotometer (Bio-Rad, Hercules, CA, USA).

### 2.5. ROS Production

The hippocampus and cortex tissues were homogenized with ice-cold saline and centrifuged at 12,000 ×g for 10 min at 4°C. The supernatant was used to detect the levels of ROS. ROS were measured using the redox-sensitive fluorescent dye, DCFH-DA. Conversion of nonfluorescent DCFH-DA to fluorescent dichlorofluorescein (DCF) in the presence of ROS was measured on a microplate reader. Fluorescence emission intensity of DCF (538 nm) was measured in response to 485 nm excitation. The level of intracellular ROS was expressed as a percentage of control cultures incubated in DCFH-DA.

### 2.6. MDA, SOD, and GSH-Px Assays

The hippocampus and cortex tissues were homogenized with ice-cold saline and centrifuged at 12,000 ×g for 10 min at 4°C. The supernatant was used to detect the levels of MDA and the activity of SOD according to the manufacturer's instructions by using Universal Microplate Spectrophotometer (Bio-Rad, Hercules, CA, USA).

### 2.7. Western Blot Analysis

The hippocampus and cortex tissues were homogenized and lysed in ice-cold RIPA buffer (containing 1 : 100 PMSF, 1 : 100 inhibitor proteases and phosphatases cocktail) for 15 min. The lysate was centrifuged at 12,000 ×g for 10 min at 4°C. The same amount of protein (30 *μ*g) was separated by SDS-PAGE analysis gel. Then the separated protein migrated to PVDF membranes and was blocked in 5% skim milk that dissolved in Tris-buffered saline-Tween-20 (TBST) for 1 h at room temperature. The membranes containing the protein were incubated with rabbit anti-Bax (1 : 1,000, number SC-526, Santa Cruz, Barbara, CA, USA), rabbit anti-Bcl2 (1 : 1,000, number 2876s, Cell Signaling Technology, Boston, MA, USA), rabbit anti-Caspase-3 (1 : 1,000, number 9662, Cell Signaling Technology, Boston, MA, USA), and mouse anti-*β*-actin (1 : 1,000, number A5441, Sigma-Aldrich, St. Louis., MO, USA) overnight at 4°C. Then the membrane was incubated with horseradish peroxidase conjugated anti-rabbit (number 7074s, Cell Signaling Technology, Boston, MA, USA) or anti-mouse (number 7076s, Cell Signaling Technology, Boston, MA, USA) IgG antibody (1 : 1,000) for 1 h at room temperature. The membrane was visualized by using a superenhanced chemiluminescence reagent (ECL; Applygen Technologies Inc., Beijing, China).

### 2.8. TUNEL Staining

Sections were washed in xylene and rehydrated through a graded series of ethanol and double-distilled water. Then, the sections were washed in PBS and incubated with 50 *μ*L TUNEL reaction mixture for 1 h at 37°C in the dark. Further incubation with 50 *μ*L converter-POD was performed at 37°C for 30 min. The sections were then rinsed with PBS and stained with DAB substrate for 10 min at room temperature. TUNEL staining was performed using the In Situ Cell Death Detection kit (Roche Diagnostics GmbH, Mannheim, Germany). Images were analyzed by using a light microscope and LEICA QWin Plus (Leica Microsystems, Wetzlar, Germany).

### 2.9. Statistical Analysis

Experimental values were given as means ± SD. The statistical analysis between two groups would be evaluated with Student's unpaired *t*-test. Statistical analysis of the data among multigroups was performed using the SPSS 19.0 statistical software. Two-way analysis of variance (ANOVA) was applied to analyze difference in data of biochemical parameters among the different groups, followed by Dunnett's significant post hoc test for pairwise multiple comparisons. Differences were considered as statistically significant at *p* < 0.05.

## 3. Results

### 3.1. Effects of STS on Learning and Memory of SCOP-Treated Mice

**Figure 2:**
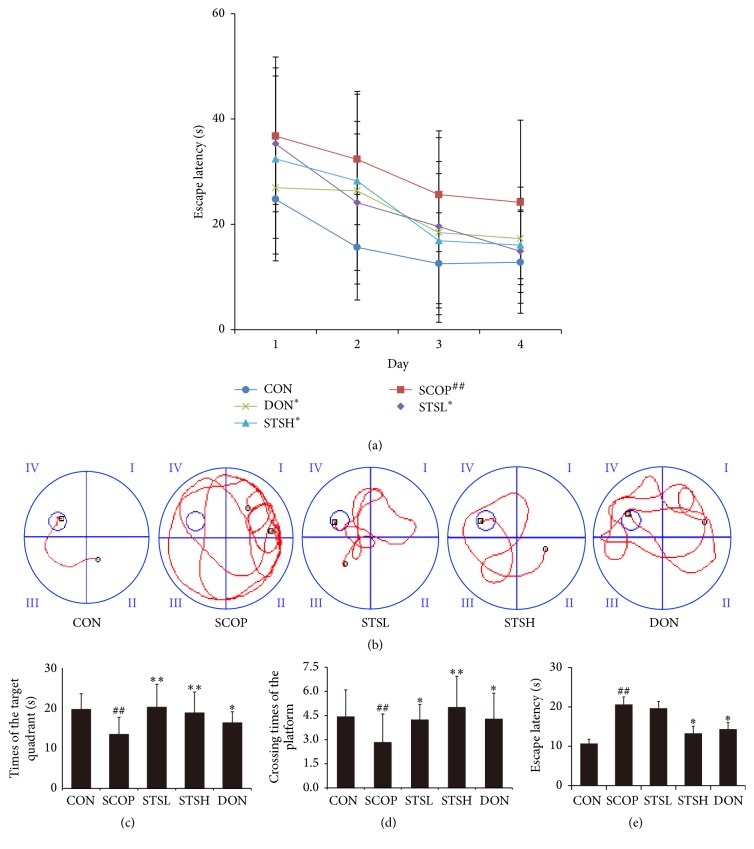
Effects of STS on learning and memory of SCOP-treated mice. (a) Escape latency of four consecutive days' test. (b) The swimming paths of respective groups on fourth day. (c) Escape latency of finding the hidden platform in the probe trial. (d) Crossing times of the target platform in the probe trial. (e) Time spent in the quadrant of the platform in the probe trial. CON: vehicle control; SCOP: scopolamine; STSL: scopolamine + STS (10 mg/kg); STSH: scopolamine + STS (20 mg/kg); DON: scopolamine + Donepezil. Data represent mean ± SEM (*n* = 10 per group). ^##^ *p* < 0.01 versus vehicle control group; ^*∗*^ *p* < 0.05 and ^*∗∗*^ *p* < 0.01 versus SCOP-treated group.

As shown in Figure 2(a), the time for mice to find the hidden platform was declined progressively during the four training days. In contrast to vehicle control group, intraperitoneal injection with SCOP remarkably increased the period of time to find the hidden platform. However, pretreatment with low (10 mg/kg) and high dose (20 mg/kg) of STS and Donepezil obviously shortened escape latency when contrasted to SCOP group. The corresponding swimming paths of each group on the fourth trail day were shown in Figure 2(b). SCOP group presented a chaotic and longer swimming path, which were improved by STS and Donepezil. On the fifth day, the probe trial was performed by removing the platform and allowing the mice to swim freely to estimate their spatial-working memory (Figures 2(c)–2(e)). SCOP group presented a longer latency (20.2 ± 2.3 s), a fewer times (2.9 ± 1.6) crossing the position of the removed platform, and a fewer times (13.4 ± 4.1 s) spent in the target quadrant, which were ameliorated by STS and Donepezil. These results demonstrated that treatment with STS remarkably reversed the cognition deficits, which was induced by SCOP.

### 3.2. Effect of STS on the Activity of AChE and ChAT in SCOP-Treated Mice

**Figure 3:**
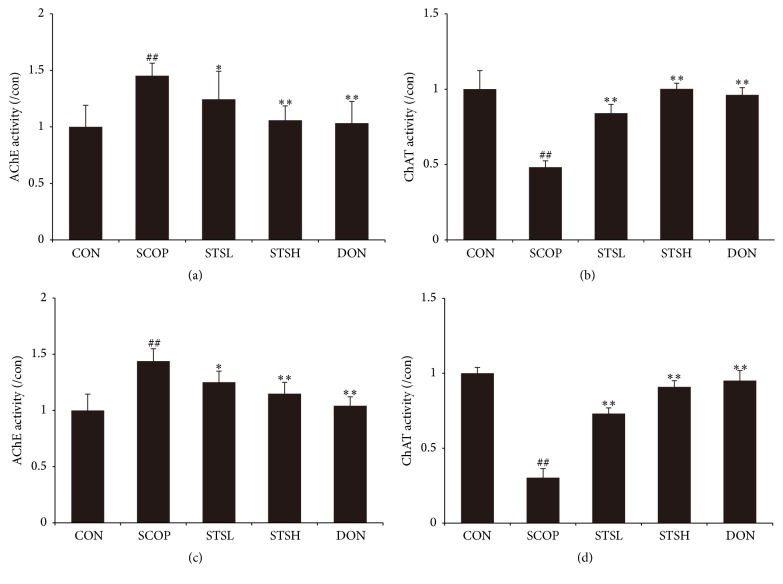
Effect of STS on the activity of AChE and ChAT in SCOP-treated mice. The supernatant of hippocampus homogenate was used for the assay of (a) AChE and (b) ChAT activities. The supernatant of cortex homogenate was used for the assay of (c) AChE and (d) ChAT activities. Data represent mean ± SEM (*n* = 10 per group). CON: vehicle control; SCOP: scopolamine; STSL: scopolamine + STS (10 mg/kg); STSH: scopolamine + STS (20 mg/kg); DON: scopolamine + Donepezil. ^##^ *p* < 0.01 versus vehicle control group; ^*∗*^ *p* < 0.05 and ^*∗∗*^ *p* < 0.01 versus SCOP-treated group.

To illuminate the potential mechanism of STS in ameliorating cognition deficiency caused by SCOP, the activities of cholinergic marker enzymes were detected. As shown in Figure 3(a), SCOP caused a remarkable increase of AChE activity in both hippocampus (1.43 ± 0.19%) and cortex (1.41 ± 0.17%), suggesting that the dysfunction of cholinergic nervous system may facilitate the process of cognitive impairment, while the treatment with STS and Donepezil significantly decreased the AChE activity. As shown in Figure 3(b), the activity of ChAT in SCOP-treated group was decreased sharply in both hippocampus (0.48 ± 0.07%) and cortex (0.27 ± 0.09%), whereas STS enhanced the activity of ChAT significantly. Thus, STS could protect against SCOP-induced dysfunction of cholinergic marker enzymes.

### 3.3. Effect of STS on the Oxidative Stress Status in SCOP-Treated Mice

**Figure 4:**
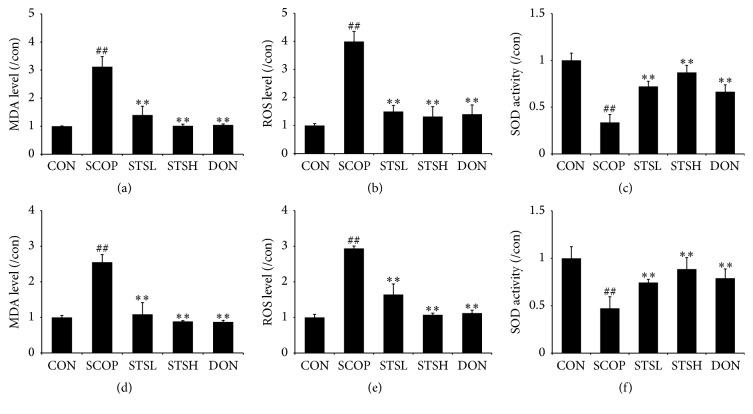
Effect of STS on the oxidative stress status in SCOP-treated mice. The supernatant of hippocampus homogenate was used for the assay of (a) ROS and (b) MDA levels and (c) SOD activity. The supernatant of cortex homogenate was used for the assay of (d) ROS and (e) MDA levels and (f) SOD activity. Data represent mean ± SEM (*n* = 10 per group). CON: vehicle control; SCOP: scopolamine; STSL: scopolamine + STS (10 mg/kg); STSH: scopolamine + STS (20 mg/kg); DON: scopolamine + Donepezil. ^##^ *p* < 0.01 versus vehicle control group; ^*∗∗*^ *p* < 0.01 versus SCOP-treated group.

Oxidative stress status was also determined in both hippocampus and cortex of SCOP-treated mice (Figures 4(a)–4(c)). ROS (4 ± 0.41% or 2.96 ± 0.05%) and MDA (3.02 ± 0.44% or 2.52 ± 0.17%) levels were robustly increased, while activity of SOD (0.38 ± 0.11% or 0.47 ± 0.16%) was suppressed in SCOP group. Both STS and Donepezil decreased the MDA and ROS levels and increased the SOD activity.

### 3.4. Effect of STS on the Protein Expressions of Bax, Bcl2, and Caspase-3 in SCOP-Treated Mice

**Figure 5:**
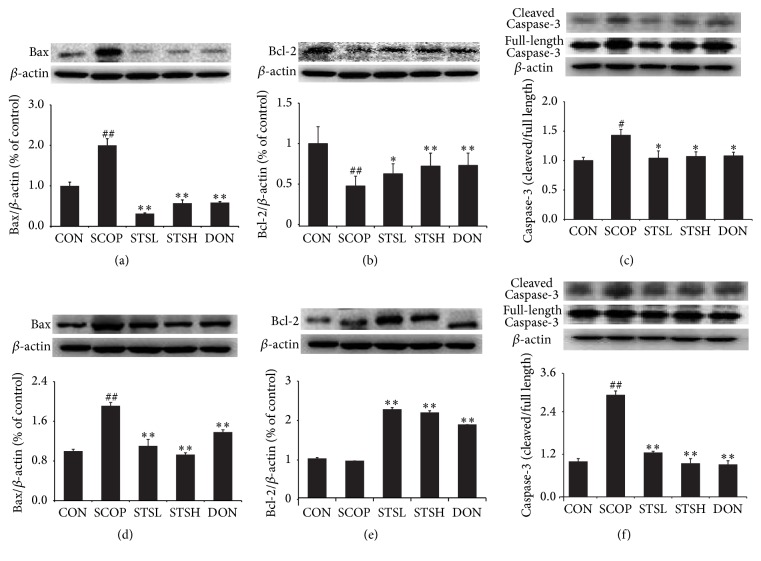
Effect of STS on the protein expressions of Bax, Bcl2, and Caspase-3 in SCOP-treated mice. Proteins expression of (a) Bax, (b) Bcl-2, and (c) Caspase-3 was detected in hippocampus. Proteins expression of (d) Bax, (e) Bcl-2, and (f) Caspase-3 was detected in cortex. CON: vehicle control; SCOP: scopolamine; STSL: scopolamine + STS (10 mg/kg); STSH: scopolamine + STS (20 mg/kg); DON: scopolamine + Donepezil. ^#^ *p* < 0.05 and ^##^ *p* < 0.01 versus vehicle control group; ^*∗*^ *p* < 0.05 and ^*∗∗*^ *p* < 0.01 versus SCOP-treated group.

As shown in Figures 5(a)–5(c), intraperitoneal injection with SCOP remarkably increased the proapoptotic proteins Bax (2 ± 0.12% or 1.82 ± 0.08%) and cleaved Caspase-3 (1.44 ± 0.07% or 2.87 ± 0.12%) expression and decreased the expression of Bcl-2 (0.48 ± 0.1% or 0.94 ± 0.02%) in both hippocampus and cortex. Both STS and Donepezil significantly upregulated the Bcl-2 expression and downregulated the Bax and cleaved Caspase-3 expressions when compared to SCOP group. These results demonstrated that STS could protect against SCOP-induced memory deficit through antiapoptosis.

### 3.5. Effect of STS on Neuronal Apoptosis in the Hippocampus

**Figure 6:**
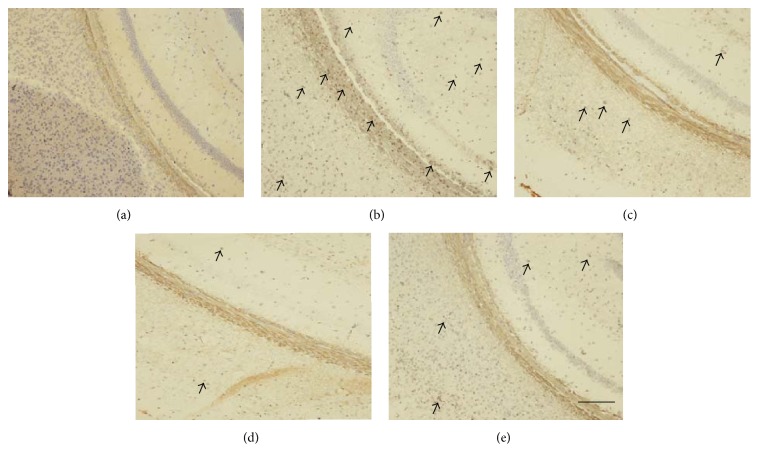
TUNEL staining in the hippocampus of SCOP-treated mice. (a) Vehicle control; (b) scopolamine; (c) scopolamine + STS (10 mg/kg); (d) scopolamine + STS (20 mg/kg); (e) scopolamine + Donepezil. Black arrows showed the neuronal apoptosis. Scale bar: 100 *μ*m.

As shown in Figure 6, TUNEL-positive cells were stained deep brown in the hippocampus. Compared with vehicle control mice, the neuronal apoptosis in the hippocampus of SCOP-treated mice was prominently increased. STS and Donepezil markedly attenuated the neuronal apoptosis in SCOP-treated mice. These results indicated that STS could protect against SCOP-induced neuronal apoptosis.

## 4. Discussion

In this study, a classical AD-like model induced by scopolamine was employed to evaluate the protective effect of STS [11]. We found that STS administration (10 mg/kg and 20 mg/kg) could improve SCOP-induced learning and memory impairment in Kunming mice. Meanwhile, STS could obviously improve central cholinergic neurotransmission and attenuate oxidative damage. In addition, STS could protect against SCOP-induced apoptosis in hippocampus and cortex.

AD is one of the most ordinary neurodegenerative diseases. Progressive impairment of cognition and affective disorder were considered as typical symptoms of AD [23]. The cognitive deficiency associated with AD is considered to be primarily related to disorder of cholinergic neurotransmission in the cerebral hippocampus and cortex [23, 24]. Various factors can induce brain impairment by influencing the synthesis, release, and uptake of acetylcholine (ACh) [25]. Scopolamine, an anticholinergic drug, can block ACh receptors and lead to a significant increase of acetylcholinesterase (AChE) level in the hippocampus and cortex [26]. The SCOP-induced amnesic model has been widely used as a pharmacological model of memory dysfunction [27]. We employed the Morris water maze test to measure the memory deficits of SCOP-treated mice. Result showed that SCOP group had a longer latency, a fewer times crossing the area of the hidden platform, and a fewer times spent in the quadrant of the original platform, which demonstrated that an amnesia model induced by SCOP was successfully established.

AChE is an important regulatory enzyme that rapidly hydrolyzes ACh, while ChAT is an enzyme that associated with the synthesis of ACh [28]. During neurotransmission, ACh is released from the presynaptic neuron into the synaptic cleft and binds to ACh receptors on the postsynaptic membrane, relaying the signal from the nerve. AChE, also located on the postsynaptic membrane, terminates the signal transmission by hydrolyzing ACh. The alterations in the membrane can be a decisive factor in changing the conformational state of the AChE molecule [25]. The results of increasing activity of AChE and decreasing activity of ChAT in the cortex and hippocampus of SCOP-treated mice were consisted with previous studies [26, 27]. Therefore, protecting cholinergic system from functional degeneration and sustaining the normal activity of ChAT and AChE might be serviceable against SCOP-induced amnesia.

STS is a sulfonated product of Tan IIA [11]. According to previous studies, STS and Tan IIA both have cardioprotective effect on cardiovascular injury. In addition, they also possess other similar pharmacological functions, such as antioxidative stress [13, 17], anti-inflammation [17, 18], and antiapoptosis [19, 20]. Interestingly, Liu et al. found that Tan IIA could remarkably ameliorate cognitive impairment in STZ-treated mice. The effects might be mediated by ameliorating the damage of cholinergic system and attenuating oxidative damage. Thus, we speculate that STS could also improve cholinergic system dysfunction. In this study, Morris water maze results showed that low dose (10 mg/kg) and high dose (20 mg/kg) of STS and Donepezil obviously shortened the escape latency, improved the swimming path, and presented more times crossing the position of the removed platform and more time spent in the target quadrant in the probe trial. Meanwhile, STS and Donepezil also significantly decreased the AChE activity and increased the ChAT activity. These results demonstrated that STS could protect against the cholinergic system dysfunction.

Imbalance between reactive oxygen species (ROS) generation and removal has been known to be involved in neuronal damage [29]. ROS can cause extensive damage to lipids, proteins, and DNA, leading to change in structure and function of neural cells in the brain [30]. Malondialdehyde (MDA) is an indicator of lipid peroxidation [31]. SOD is an antioxidant enzyme, inducing increased free radical generation [32]. In this study, treating with SCOP robustly increased ROS and MDA levels and suppressed the activity of SOD. Evidence shows that STS could be used as an antioxidant to validly inhibit the formation of reactive oxygen radicals [33] and eliminate lipid-free radicals [34]. The present study showed that STS conspicuous increased SOD activity and reduced MDA and ROS levels in both hippocampus and cortex. These results indicated that STS could upregulate oxidation tolerance in hippocampus and cortex.

Apoptosis is considered to be one of the main causes of neurodegeneration. Oxidative stress can lead to the consecutiveness response apoptosis straightly [35, 36]. Caspase-3, which belongs to the subgroup of Caspase protease in the Caspase family, is the terminal executing enzyme in apoptosis. And it can cut the structural protein of cells and lead to apoptosis [37]. Bcl-2 can be extracted from B cell lymphoma and present a distinct capacity of antiapoptosis [38]. In contrast to Bcl-2, Bax protein exerts the opposite effect of promoting cell apoptosis [39]. Therefore, we can determine cell survival according to relative ratio of proapoptotic protein Bax and antiapoptotic protein Bcl-2. Meanwhile, several neurodegenerative diseases are associated with activation of apoptosis. Our results showed that STS remarkably downregulated the apoptotic index Bax/Bcl2 and cleaved Caspase-3 expressions in hippocampus and cortex of SCOP-treated mice. In addition, TUNEL staining showed that STS markedly attenuated the neuronal apoptosis in SCOP-treated mice. These results suggested that the protective effect of STS against SCOP-induced injury is related to inhibiting the damage induced by free radicals and consecutive apoptosis.

In conclusion, our study suggested that the cognitive-protecting activities of STS on SCOP-induced memory impairment might result from its effect of improving the cholinergic nervous system and antioxidative stress. However, the effect of STS on other AD pathological symptoms, such as synaptic degeneration, neuroinflammation, and neurite degeneration, remains unclear, which is worthy to investigate in the future.
